# The Expanding Therapeutic Armamentarium for Mitral Regurgitation: Surgical and Transcatheter Interventions

**DOI:** 10.3390/biomedicines14071539

**Published:** 2026-07-09

**Authors:** Argyro Kalompatsou, Dimitris Tousoulis, Charilila-Loukia Ververeli, Ioannis Kachrimanidis, Yannis Dimitroglou, Sotirios Tsalamandris, Maria Drakopoulou, Konstantinos Aznaouridis, Kyriakos Dimitriadis, Markos Koukos, Aggelos Papanikolaou, Vasilis Lozos, Konstantinos Toutouzas, Konstantinos Tsioufis, Constantina Aggeli

**Affiliations:** 1 First Cardiology Department, Hippokration General Hospital, National and Kapodistrian University of Athens, 11527 Athens, Greece; iro.kalompatsou@gmail.com (A.K.); harilila_med@hotmail.com (C.-L.V.); marckoukos@yahoo.gr (M.K.); agepap25@otenet.gr (A.P.);; 2 Department of Cardiothoracic Surgery, Hippokration Hospital, 11527 Athens, Greece

**Keywords:** mitral regurgitation, mitral therapies

## Abstract

**Background:** Mitral regurgitation (MR) is one of the most prevalent valvular heart diseases, with a rising global incidence. The 2025 European Society of Cardiology (ESC) guidelines introduced updated pathophysiological and morphological concepts for secondary MR, distinguishing ventricular and atrial mechanisms. Concurrent advances in cardiovascular imaging and therapeutic technologies have transformed the diagnostic and management landscape of MR. **Methods:** This review summarizes current evidence on the diagnosis and treatment of MR, with a focus on the updated ESC classification, multimodality cardiovascular imaging, minimally invasive surgical techniques, and contemporary transcatheter repair strategies. Recent literature was evaluated to highlight advances in anatomical assessment and individualized therapeutic approaches. **Results:** Multimodality imaging provides comprehensive evaluation of mitral valve anatomy, ventricular remodeling, and disease mechanisms, enabling accurate patient selection and procedural planning. Surgical management has evolved from conventional repair or replacement to minimally invasive approaches, including video-assisted right thoracotomy and robotic-assisted surgery, which have demonstrated favorable perioperative and clinical outcomes. In parallel, transcatheter interventions have expanded the therapeutic armamentarium for patients at high surgical risk or with complex anatomy. These include direct and indirect annuloplasty, transcatheter edge-to-edge repair, and emerging catheter-based repair technologies targeting specific structural abnormalities of the mitral valve apparatus. **Conclusions:** Contemporary management of MR requires an integrated understanding of disease pathophysiology, advanced imaging, and patient-specific anatomical characteristics. The combination of minimally invasive surgical techniques and rapidly evolving transcatheter interventions has broadened treatment options and supports a tailored, multidisciplinary approach to improve clinical outcomes and expand access to effective therapy for patients with severe MR.

## 1. Introduction

Mitral regurgitation (MR) is one of the most common cardiac valve diseases; recently, its prevalence has been increasingly becoming a globally endemic heart disease. In the PREVUE VALVE study, which evaluated the prevalence of valvular heart disease in a representative U.S. population aged 65–85 years, the weighted prevalence of moderate valvular heart disease was 8.2%, with higher rates among older age groups. Among the valvular lesions, the estimated prevalence of moderate or greater MR was 2%, while tricuspid regurgitation and aortic stenosis were the most common overall [1]. It affects more than >6% of those aged >65 years and up to 10% for chronic heart failure (HF) patients presenting with severe MR [2]. The etiology is heterogeneous, including primary MR (PMR), secondary MR (SMR), or mixed cases involving combined mechanisms. Untreated severe MR is associated with poor outcomes mainly due to chronic volume overload consequences leading to adverse cardiac remodeling.

**Figure 1:**
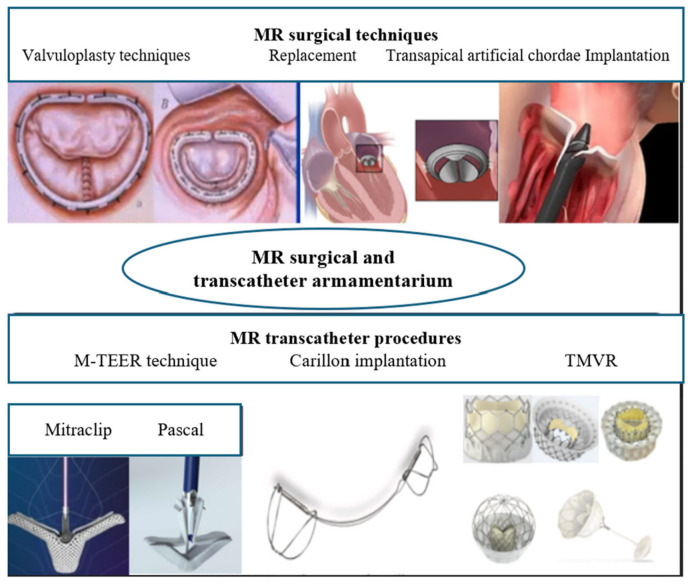
**Surgical and transcatheter treatment options for mitral valve regurgitation.** The **upper** panel illustrates the principal surgical approaches for mitral valve regurgitation, including mitral valve repair (valvuloplasty with leaflet resection and annuloplasty ring implantation), surgical mitral valve replacement, and transapical artificial chordae implantation. The **lower** panel depicts the main transcatheter treatment strategies, including Transcatheter Edge-to-Edge Repair (TEER) using the MitraClip or PASCAL system, indirect annuloplasty with the Carillon device, and transcatheter mitral valve replacement (TMVR).

Novel therapeutic options for MR patients have expanded from surgical mitral valve replacement/repair to percutaneous transcatheter procedures (Figure 1) [3]. The Mitral Transcatheter Edge-to-Edge Repair (M-TEER) has been the most commonly employed approach. Robust evidence has demonstrated the safety and efficacy of M-TEER in high-surgical-risk patients, resulting in upgraded recommendations in both European and American guidelines [4,5]. Interestingly, for those high surgical risk patients with complex mitral valve (MV) anatomy, transcatheter mitral valve replacement (TMVR) has emerged as a therapeutic alternative option [6]. The precise, modern, and accurate evaluation of MV disease, other concominant valvular abnormalities, and relevant comorbidities should be considered by the Heart Valve Team to guide optimal patient management [7].

**Etiology of MR and cardiac adaptation in acute and chronic disease settings**

Mitral regurgitation develops when the mitral valve fails to achieve effective systolic closure, allowing retrograde blood flow from the left ventricle (LV) to the left atrium (LA). Because normal MV competence depends on the integrated function of the annulus, valve leaflets, chordae tendineae, papillary muscles, LV myocardium, and LA, abnormalities affecting any of these components may result in MR [8,9,10]. In contemporary clinical practice, MR is broadly categorized into PMR and SMR, a distinction that reflects different pathophysiological mechanisms, remodeling patterns, and therapeutic implications [5,9,10].

Primary MR is caused by intrinsic structural abnormalities of the MV apparatus. In developed countries, the most common etiology is degenerative MV disease, usually related to myxomatous degeneration of the leaflets and chordal apparatus, which may ultimately lead to leaflet prolapse or flail [8,11]. Degenerative disease spans a broad morphological spectrum. Fibroelastic deficiency is typically characterized by thin leaflet tissue, focal prolapse, and chordal rupture, whereas Barlow’s disease is associated with diffuse myxomatous degeneration, redundant leaflet tissue, annular enlargement, and multisegmented prolapse [12,13]. Additional causes of PMR include rheumatic valve disease; infective endocarditis; congenital cleft lesions; papillary muscle rupture following myocardial infarction; and severe mitral annular calcification (MAC), which is particularly relevant in elderly and multimorbid patients and may contribute to restricted leaflet motion, annular dysfunction, and complex mitral valve anatomy [14].

By contrast, SMR occurs in the absence of primary structural leaflet disease and reflects failure of leaflet coaptation secondary to remodeling of the ventricle, the atrium, or both. In this setting, the valve leaflets are often morphologically preserved, but geometric distortion of the surrounding apparatus renders the valve incompetent. Several mechanisms may contribute, including LV dilatation, papillary muscle displacement, annular enlargement, regional or global LV dysfunction, and LV dyssynchrony, all of which alter the balance between closing forces and tethering forces acting on the leaflets [9,10]. Although SMR has historically been regarded predominantly as a ventricular disorder, contemporary concepts have refined this framework. The 2025 ESC Guidelines for the management of valvular heart disease distinguish ventricular SMR from atrial SMR, recognizing that these phenotypes arise from different structural substrates and may carry distinct prognostic and therapeutic implications [5]. Ventricular SMR is primarily driven by ischemic or non-ischemic LV remodeling, whereas atrial SMR is more closely linked to LA enlargement, annular dilatation, atrial fibrillation, and often heart failure with preserved ejection fraction despite relatively preserved LV geometry [5,9,10,15].

The hemodynamic consequences of MR vary substantially according to whether the lesion is acute or chronic. In acute MR, regurgitation develops abruptly before the LA and LV have had sufficient time to adapt to the sudden increase in volume load. As a result, even a relatively limited regurgitant volume may lead to a marked rise in LA pressure, acute pulmonary congestion, and hemodynamic instability. Acute severe MR, therefore, constitutes a medical emergency and may occur in settings such as chordal rupture, papillary muscle rupture after acute myocardial infarction, or acute infective valve destruction [8,11,16]. In chronic MR, by contrast, the volume overload is imposed gradually, allowing compensatory remodeling of the left-sided chambers. The LV typically undergoes eccentric enlargement in an attempt to preserve forward stroke volume, while the LA progressively dilates and becomes more compliant, thereby partially buffering the rise in filling pressures. This compensatory phase may delay symptom onset, particularly in chronic degenerative MR; however, persistent regurgitant burden eventually becomes maladaptive, promoting progressive LV dysfunction, worsening LA remodeling, atrial fibrillation, pulmonary hypertension, right-sided involvement, and ultimately symptomatic heart failure [9,10,15,16].

Importantly, the remodeling response differs between PMR and SMR. In chronic PMR, the regurgitant lesion is the primary abnormality and chamber enlargement occurs mainly as a consequence of long-standing volume overload. In SMR, however, the relationship is bidirectional: ventricular or atrial remodeling generates the regurgitation, while the regurgitant volume further increases preload, wall stress, annular deformation, and chamber enlargement, thereby aggravating the underlying disease process [9,10,15]. In this way, SMR should be viewed not merely as a marker of advanced cardiomyopathy, but also as an active contributor to progressive heart failure. Accordingly, a mechanistic classification of MR is not simply descriptive; it has direct implications for prognosis, timing of intervention, and the selection of surgical-versus-transcatheter treatment strategies [5,9,10,15,16].

**The role of cardiovascular imaging in the evaluation of MR patients**

Cardiovascular imaging is central to the evaluation of MR, as it informs diagnosis, severity grading, mechanistic classification, procedural planning, and follow-up. Beyond confirming the presence of MR, imaging should define the mechanism of regurgitation, characterize the morphology of the MV apparatus, quantify the hemodynamic burden of disease, and assess the extent of cardiac remodeling, all of which directly influence therapeutic decision-making [17].

Transthoracic echocardiography (TTE) remains the first-line imaging modality and the cornerstone of MR assessment. It provides essential information regarding leaflet morphology and motion, annular dimensions, chamber size, ventricular systolic function, pulmonary pressures, and associated valvular lesions. Importantly, MR severity should be evaluated using an integrated multiparametric approach, combining quantitative indices such as effective regurgitant orifice area (EROA), regurgitant volume, regurgitant fraction, vena contracta, and pulmonary vein flow, along with qualitative features, MV anatomy, and the patient’s hemodynamic status, since isolated parameters may be misleading in eccentric, multiple, or dynamic regurgitant jets [17].

When transthoracic imaging is inconclusive or when intervention is being considered, transesophageal echocardiography (TEE) offers superior anatomical definition of the MV apparatus, including flail segments, commissural lesions, clefts, annular calcification, and subvalvular abnormalities. In parallel, three-dimensional (3D) echocardiography, particularly 3D TEE, has improved the anatomical characterization of MR by enabling en face visualization of the valve, more accurate scallop localization, and better assessment of annular geometry and leaflet coaptation. These features are particularly relevant for procedural planning in repair-oriented strategies such as M-TEER. Emerging applications, including patient-specific virtual 3D mitral models derived from TEE datasets, may further enhance preprocedural planning in complex cases [16,18]. In addition, left atrial strain has emerged as a promising adjunctive marker of early atrial dysfunction and may provide incremental information in chronic MR, although its role in routine clinical decision-making remains to be fully established [15,19].

Cardiovascular magnetic resonance (CMR) is the most important complementary modality when echocardiographic findings are discordant with symptoms, ventricular remodeling, or overall clinical impression. CMR allows highly reproducible quantification of left ventricular volumes, regurgitant volume, and regurgitant fraction, and is particularly useful in patients with eccentric jets, multiple jets, poor acoustic windows, or borderline MR severity. In addition, it provides tissue characterization through late gadolinium enhancement (LGE), thereby refining the assessment of myocardial disease burden, especially in secondary MR [20]. By contrast, positron emission tomography (PET) currently has only a limited, selective role and should be viewed as an adjunctive modality in highly selected scenarios rather than part of routine MR evaluation [21]. Overall, a multimodality imaging strategy should be individualized according to the MR mechanism, disease stage, and the anticipated therapeutic pathway.

**Updates from the 2025 ESC Guidelines, Comparison with the ACC/AHA Guidelines, and the Role of Optimal Medical Therapy**

Patients with chronic severe PMR and impaired LV function should receive guideline-directed medical therapy (GDMT) according to the HF Guidelines [22]. In an acute setting, afterload reduction with sodium nitroprusside has been used as a bridge to an intervention. Inotropic agents and diuretics are usually indicated to reduce filling pressures, while implantation of an intra-aortic balloon pump may further reduce afterload in exceptional cases of acute PMR.

Furthermore, in patients with ventricular SMR, GDMT for HF is primarily recommended prior to any MV intervention when required. The combination of ACE-Is/ARBs or angiotensin receptor/neprilysin inhibitors, beta-blockers, mineralocorticoid receptor antagonists, and sodium-glucose co-transporter 2 inhibitors (SGLT2i) at the maximum tolerated doses is recommended according to the HF Guidelines [22]. Cardiac resynchronization therapy (CRT) should also be considered as part of HF management before an MV intervention, when appropriate [23]. It is worth noting that none of the trials systematically integrated the current “four pillars” of HF treatment (ACEI/ARB/ARNI, beta-blockers, mineralocorticoid antagonists, and SGLT2 inhibitors) across all patients. Additionally, the role of GDMT in atrial SMR is also poorly understood. More trials are required to determine the role of contemporary therapy in the clinical setting, whether used alone or as part of combined therapy, in the context of widespread MR etiology.

The 2025 ESC and 2020 ACC/AHA guidelines both recommend surgery for symptomatic patients with severe PMR and for asymptomatic patients with evidence of LV dysfunction, defined by a left ventricular ejection fraction (LVEF) < 60% and/or an LV end-systolic diameter (LVESD) > 40 mm. The ESC guidelines additionally include an indexed LVESD threshold > 20 mm/m^2^ to account for body size. In patients at high or prohibitive surgical risk, both guidelines support M-TEER (Class II recommendation). A notable distinction is that the ACC/AHA guidelines encourage referral to experienced Heart Valve Centers to consider early intervention in selected patients, whereas the ESC guidelines continue to rely primarily on echocardiographic thresholds of LV remodeling and dysfunction to guide intervention timing.

For SMR, both the 2025 ESC and 2020 ACC/AHA guidelines emphasize optimizing GDMT for HF before considering MV intervention. CRT should also be implemented in eligible patients. The ACC/AHA guidelines assign a Class IIa recommendation to M-TEER in patients who fulfill the COAPT trial criteria despite optimal medical therapy. In contrast, the 2025 ESC guidelines have upgraded M-TEER to a Class I recommendation for COAPT-like patients, reflecting the growing body of evidence supporting its clinical benefit. The ESC guidelines further retain a Class IIb recommendation for M-TEER in carefully selected non-COAPT patients who remain symptomatic despite optimized medical therapy, primarily to improve symptoms and quality of life. In patients with advanced HF, both guidelines recognize the role of advanced HF therapies, including durable left ventricular assist devices and heart transplantation, when clinically appropriate.

The management of atrial SMR differs between the two guidelines. The 2020 ACC/AHA guidelines assign a Class IIb recommendation to mitral valve surgery in selected patients. In contrast, the 2025 ESC guidelines advocate a more comprehensive, mechanism-based approach that emphasizes treatment of the underlying atrial cardiomyopathy. In symptomatic patients with severe atrial SMR despite optimal medical therapy, MV surgery, concomitant surgical atrial fibrillation ablation (when indicated), and left atrial appendage occlusion are assigned a Class IIa recommendation. M-TEER is considered a Class IIb option for carefully selected symptomatic patients who are not suitable candidates for surgery.

## 2. Surgical Procedures

### 2.1. Surgical Mitral Valve Repair

Degenerative MV disease encompasses a wide spectrum of lesions that require a wide variety of surgical techniques for repair [24]. Several surgical approaches for accessing the MV have been described, highlighting the hot-topic issue [25]. Cardiovascular imaging plays a crucial role in determining the type of surgical procedure [26].

While median sternotomy remains the most popular approach, several surgeon groups have significantly transformed the incision into a lower hemisternotomy. Adopted non-sternotomy approaches, also known as video-assisted approaches, including right thoracotomy and robotic surgery, have been associated with more favorable surgical outcomes, including shorter hospital stay, fewer postoperative complications, and lower in-hospital mortality [27]. However, its high cost is a significant barrier to widespread clinical implementation [28].

Although valve replacement has historically been favored, several studies, due to concerns regarding tissue fragility, surgical complexity, and surgical risk, have shifted toward MV repair (MVr) as a safe and durable surgical option. Surgical repair techniques include resection of prolapsed or flail leaflet segments, implantation of artificial chordae (neo-chordae) to restore leaflet support, annuloplasty with a prosthetic ring to restore the mitral annulus contraction and shape, and commissuroplasty to adjust leaflet coaptation (Figure 1). Even extensive anterior prolapse can be effectively repaired with a triangular resection [29].

Surgical repair with ring or band annuloplasty is among the most comprehensive treatments for ischemic MR. Although this approach is standard of care, long-term durability of ischemic MR repair is not ideal and can result in unintended failures due to procedural errors, leading to device dislodgement and dehiscence [30].

**Figure 2:**
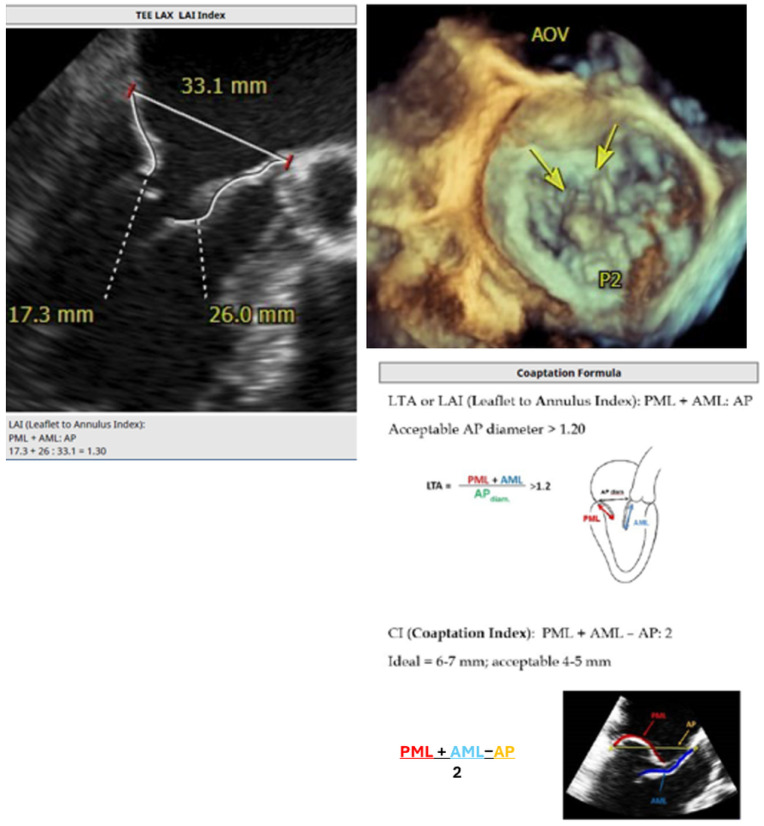
Schematically, the indices used in NeoChord’s procedure. 3D zoom of the patient’s mitral valve showing prolapse and flail at the P2 segment, with favorable indices predicting a good postprocedural result. The leaflet-to-annulus index and the coaptation index are presented as favorable anatomical data.

**Figure 3:**
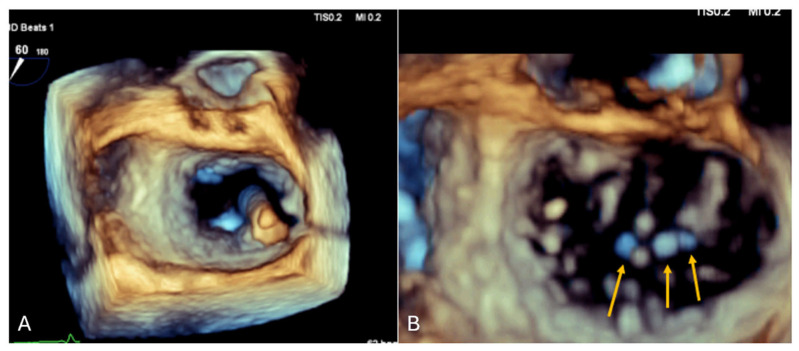
Transapical NeoChord’s procedure implantation: (**A**) 3D mitral valve zoom from left atrium perspective. Open the device in the left atrium, capturing the posterior leaflet in order to perform the chord implantation. (**B**) After 3-chord implantation (3 arrows) at the P2 prolapsing area (**B**).

In the setting of a very limited or normal leaflet tissue, it may be preferred to avoid leaflet resection and preserve native leaflet tissue while restoring physiological function. The concept of “respect rather than resect” of MV surgical repair was introduced as an alternative to leaflet resection techniques [31,32]. In the setting of very limited or normal leaflet tissue, proceed with a chordal transfer or surgical techniques using polytetrafluoroethylene (PTFE) (i.e., loop technique, loop-in-loop technique, or single neochordoplasty). Transapical off-pump neochordal implantation is a minimally invasive surgical technique for MVr in patients with PMR, particularly degenerative MR due to leaflet prolapse or flail. These chordae (NeoChord DS1000, Harpoon, Louis Park, MN, USA) are attached to the prolapsed leaflet and then tensioned on the beating heart under TEE guidance to restore valve competency (Figure 2 and Figure 3) [33,34]. Clinical studies on transapical neochordae implantation technique have demonstrated excellent early-, mid-, and long-term outcomes, ideally in patients with isolated posterior prolapse [35,36]. Similarly, our institutional experience demonstrates that transapical NeoChord implantation in patients with favorable MV anatomy is a safe procedure, yielding durable mid-term outcomes at 20 months of follow-up, characterized by low cardiac mortality and high freedom from both reoperation and rehospitalization [37,38].

In ventricular SMR, novel techniques have emerged to indirectly repair the mitral apparatus by combining left ventricular aneurysmectomy with patch reconstruction [39,40].

According to the MITRACURE trial [41], degenerative MR patients continued to be referred late in the disease course. The MVr rate was 80%, with a 6% intraoperative repair failure rate. Repair rates declined with age, comorbidities, and complex anatomy, and they increased with center volume.

### 2.2. Surgical Mitral Valve Replacement

Surgical MV replacement is avoided over repair for many reasons, especially in patients with degenerative MV disease [38]. The advantages of surgical MVr include lower perioperative risk, improved event-free survival for surgical patients, freedom from the various complications of prosthetic heart valves, and better postoperative LV function [42,43]. However, it remains fairly prevalent among patients with complex lesions. This situation is reversed for patients with rheumatic disease, for whom MV replacement (MVR) rates are as high as 50% in reference centers [44]. While prosthesis selection seems clear for elderly and young patients, no data indicate a significant survival advantage of mechanical over bioprosthetic valves, or vice versa, in middle-aged patients. According to the most recent guidelines on valvular heart disease management, mechanical prostheses are considered reasonable based on the patient’s informed preference, provided there are no contraindications to anticoagulation.

## 3. Transcatheter Procedures

### 3.1. Transcatheter Mitral Valve Repair

Given the complexity of the MV apparatus, it is useful to consider transcatheter approaches according to the major structural abnormalities they address. Recent advances in transcatheter MVr have enriched the interventional armamentarium, offering new options for challenging MV anatomy.

The treatment of SMR typically involves a multi-step approach with GDMT or CRT, which may reduce MR severity, improve symptoms, reduce hospitalizations, and prolong survival. Percutaneous annuloplasty therapies are intended to replicate the surgical procedure by supporting the annulus and preventing further dilatation when this is the main mechanism of MR. New generations of annuloplasty rings are designed to combine structural support with enhanced flexibility, allowing for better adaptation to the dynamic nature of the mitral annulus.

#### 3.1.1. Indirect Annuloplasty

Carillon Mitral Contour System: The Carillon Mitral Contour System is an indirect percutaneous mitral annuloplasty device implanted via the coronary sinus for the treatment of SMR. It uses the anatomic proximity of the coronary sinus and great cardiac vein to the posterior part of the mitral annulus. The system consists of a nitinol shaping ribbon positioned between two anchors that can be tensioned to reduce mitral annular dimensions and enhance leaflet coaptation [45]. With ongoing large-scale randomized studies such as the EMPOWER trial, stronger evidence on survival and long-term outcomes will soon be available.

Cerclage annuloplasty: The transcatheter mitral cerclage ventriculoplasty [46] should be an alternative therapy for patients with advanced HF and persistent SMR. Cerclage led to a significant reduction in MR and appeared to prevent further cardiac chamber dilation in subjects ill-suited or ineligible for TEER, and further led to substantial improvements in quality of life, particularly in non-ischemic cardiomyopathy. Although some patients developed heart block requiring pacemakers, there were no procedural deaths, strokes, or major vascular complications.

#### 3.1.2. Direct Annuloplasty

Cardioband: The Cardioband is an incomplete adjustable Dacron band, similar to a surgical semirigid band that is implanted supra-annularly along the posterior annulus from the anterolateral to posteromedial commissures. The Cardioband is most suitable for patients with SMR, Carpentier type I (annular dilation/deformation) and type IIIB (symmetrical leaflet tethering) MR, with a large left ventricular end-diastolic diameter (70 mm). It is a device designed to perform direct percutaneous annuloplasty (supra-annular fixation, like in surgery) of symptomatic patients (NYHA III-IV) with dilated cardiomyopathy and moderate–severe SMR (due to mitral annulus enlargement) by means of a half-ring implanted in the posterior mitral annulus, with beating heart, and under fluoroscopic and TEE guidance [45]. Similar to the Carillon, an important feature is that it allows further interventions at the leaflet level (e.g., edge-to-edge clipping) or may serve as a rescue procedure.

Mitralign: The Mitralign Percutaneous Annuloplasty System is a direct percutaneous annuloplasty system. It uses radiofrequency energy to penetrate sutures for two bident pledgets into the mitral annulus tissue posterior and anterior to the commissure (both atrial and ventricular sides). Symptomatic severe SMR patients with reduced LVEF on GDMT may be considered as the target group [45].

#### 3.1.3. Transcatheter Edge-to-Edge Repair

**Figure 4:**
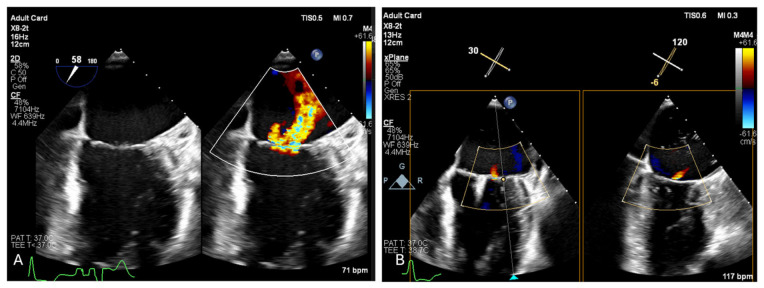
Successful implantation of a MitraClip device. (**A**) Severe secondary mitral regurgitation in a patient with ischemic cardiomyopathy. (**B**) Trivial residual mitral regurgitation after deployment of the MitraClip device.

**Figure 5:**
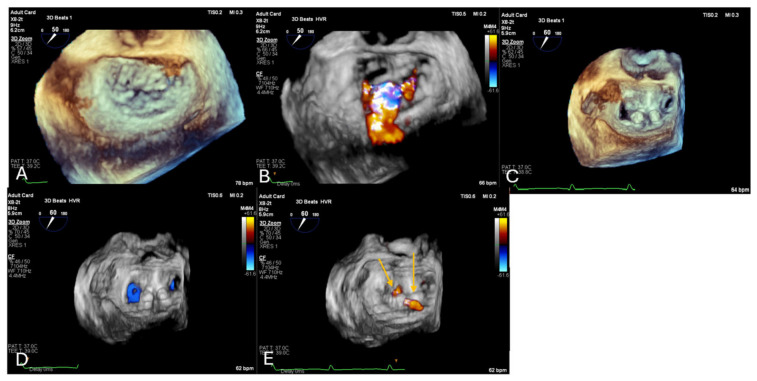
Severe secondary mitral regurgitation. 3D zoom from the left atrium perspective (**A**) with Color Doppler (**B**). After 2 PASCAL device implantations, 2 new mitral valve orifices were demonstrated (**C**,**D**) with trivial residual mitral regurgitation ((**E**)-arrows).

Lately, advanced novel transcatheter techniques have expanded the “therapeutic option” into a new era. Alfieri first described the surgical repair of severe MR using an edge-to-edge technique [47,48], and the M-TEER technique mimicked this by an interventional device implantation for patients at high surgical risk or who were inoperable [49]. The currently widely available commercial devices are the MitraClip (Abbott, Santa Clara, CA, USA) and the PASCAL (Edwards Lifesciences, Irvine, CA, USA) (Figure 4 and Figure 5). The DragonFly transcatheter MVr system (Hangzhou Valgen Medtech Co., Shenzhen, Guangdong, China) received the European Conformity (CE) marking in April 2025 [50].

In patients with severe symptomatic PMR who are at high or prohibitive surgical risk, M-TEER should be considered in anatomically suitable candidates following evaluation by a multidisciplinary Heart Team (Class IIa recommendation) [5].

Additionally, M-TEER is recommended as Class I in high-surgical-risk symptomatic patients with severe SMR despite optimal GDMT and without concomitant coronary artery disease. Finally, M-TEER may be considered in symptomatic severe atrial SMR not eligible for surgery (Class IIb) [5].

The MitraClip system received CE mark approval in 2008 and has since then known a steady growth in its use [51,52,53,54]. Approval by the US Food and Drug Administration (FDA) was granted after the results of the EVEREST II trial were published for PMR and after presentation of the COAPT trial for SMR [54,55]. The fifth-generation (G5) device was introduced in 2025 and includes four device sizes to provide tailored solutions for varying anatomies: NT (9 mm arm length; 4 mm arm width), NTW (9 mm arm length; 6 mm arm width), XT (12 mm arm length; 4 mm arm width), and XTW (12 mm arm length; 6 mm arm width) (Figure 4) [56]. The longer clip arms (XT/XTW) allow for treatment of larger coaptation gaps and leaflet flails beyond the strict anatomic and morphologic EVEREST inclusion criteria.

The PASCAL system received its CE mark approval in 2019, providing an alternative M-TEER platform with distinct features. It currently features two different devices, the PASCAL P10 and the PASCAL ACE. The PASCAL P10 has a 5 mm central spacer with 10 mm wide and 9 mm long paddles, which are grasped through a spring-loaded mechanism, the clasps (Figure 5) [56]. The PASCAL device received its FDA approval for treatment of PMR in 2022, according to the results of the CLASP IID trial, which demonstrated non-inferiority of PASCAL versus MitraClip for primary safety and effectiveness [57].

Regarding the DRAGONFLY system, 2-year outcomes in China and 6-month outcomes in European centers were recently presented. The 1-year safety and efficacy of the device have been shown in the DRAGONFLY-DMR trial for patients with degenerative MR [50]. A novel M-TEER system has recently been introduced, with first-in-human experience and 1-year outcomes in patients with SMR reported. The SQ-Kyrin-M device, specifically designed for SMR, further supports the safety and effectiveness of TEER in contemporary clinical practice [58].

Eligibility criteria for the M-TEER technique: anatomical inclusion criteria had already been integrated for both PMR and SMR patients in the early EVEREST trial, defining a patient population with a high likelihood of an optimal M-TEER result. Anatomical exclusion criteria in EVEREST II were an MV orifice area of <4.0 cm^2^, flail gap of ≥10 mm, flail width of ≥15 mm, and leaflet tethering with a coaptation gap of >11 mm. Beyond these EVEREST MV criteria, the complexity of the M-TEER procedure increases, incorporating patients with more complex anatomy and more advanced HF disease. Additionally, in the EXPAND registry, anatomical complexity was defined as follows: wide coaptation gap (≥15 mm), large flail gap (≥10 mm), jet outside the anterior 2/posterior 2 (A2/P2), small mitral valve area (MVA), or calcification. Consequently, patients meeting unfavorable anatomical and functional criteria lead to less optimal results. Identified additional anatomical quantitative predictors of procedural success include the coaptation leaflet reserve [59], the leaflet-to-annulus index [60], and asymmetrical tethering [61]. Contraindications should be carefully evaluated, as severe calcification within the grasping zone, active endocarditis, and hemodynamically significant mitral stenosis constitute absolute contraindications to M-TEER.

So far, several discussions have taken place regarding the diversities of the first trials, the MITRA-FR and COAPT, in post-procedural outcomes [62]. In the COAPT trial, M-TEER significantly reduced the incidence of HF rehospitalization and improved survival in a selected population of patients with SMR compared with GDMT alone, with a prognostic benefit that was maintained at long-term follow-up. COAPT patients in whom the MR was seen as “disproportionate” to the degree of LV impairment had superior post M-TEER outcomes. This concept should be extended to atrial SMR patients with “disproportionate” MR patients having a higher degree of MR compared to left atrial volume. It seems that the M-TEER technique had no favorable results concerning symptoms and 2-year mortality, but it should be an option for high surgical-risk patients [63]. In the real world, patients with atrial SMR are older, with several comorbidities and MAC, resulting in the M-TEER technique being the only interventional option.

It is worth noting that among patients who underwent M-TEER for SMR in the TVT Registry, nearly one-half would have been ineligible for the COAPT trial. Health status improvement at 30 days was similar in COAPT-ineligible and COAPT-eligible patients, but trial-ineligible patients had higher 1-year rates of death or HF hospitalization [64]. In the EXPANDed studies, COAPT-like patients experienced significant improvements in MR, HF hospitalizations, and quality of life at 1 year, comparable to those of non-COAPT-like patients. These findings suggest that M-TEER may be an effective therapeutic option in appropriately selected patients outside COAPT eligibility [65].

Recent randomized trials, most notably RESHAPE-HF2 and MATTERHORN, have expanded the evidence base supporting Μ-TEER. RESHAPE-HF2 demonstrated that M-TEER significantly reduces HF hospitalizations and cardiovascular death in well-selected patients with moderate-to-severe SMR, especially those with recent decompensation and favorable MV anatomy. MATTERHORN confirmed the noninferiority of M-TEER compared to surgery in high-risk patients, with a markedly better safety profile [66]. It is important to highlight that a post hoc analysis of the MATTERHORN trial in the atrial SMR subgroup showed lower event rates of the M-TEER technique compared with surgical treatment. These findings further support the non-inferiority of M-TEER with respect to mortality and rehospitalization outcomes [67]. The retrospective MITRA-TUNE registry [68] demonstrated that M-TEER was safe and effective in achieving a significant reduction of MR in patients with atrial SMR, leading to positive LA and mitral annular reverse remodeling.

Collectively, COAPT, MITRA-FR, RESHAPE-HF2, and MATTERHORN indicate that the success of M-TEER depends less on MR severity alone than on appropriate patient selection, optimization of GDMT, ventricular remodeling, anatomical suitability, and overall clinical phenotype. Current evidence therefore supports a Heart Team-based, individualized approach rather than universal application of transcatheter intervention.

*M-TEER technique as a bridge to transplantation:* Patients awaiting heart transplantation (HTx) often need bridging therapies to reduce worsening and progression of the underlying disease. In the first report from the MitraBridge registry, MitraClip as a bridge to HTx proved to be an effective treatment strategy at 1 year for patients with advanced HF who were potential candidates for HTx [69]. After 2 years of follow-up, the use of MitraClip in this group of patients allowed for elective HTx or transplant eligibility in one-third of patients, with no need for transplantation in 22.5% of cases [70].

M-TEER procedures have been extended to additional, more complex situations. In this direction, small studies on specific populations, as well as case reports [66] involving complex anatomy or novel techniques, have been published. In that regard, assessment of the feasibility and safety of M-TEER in cardiogenic shock is of utmost importance. Furthermore, the combination of VV-ECMO support and M-TEER represents a viable therapeutic alternative in an acute setting. In addition, it has recently been proposed that an organized “mobile m-TEER team” may be an attractive strategy for timely, safe, and sustainable acute MR reduction in high-risk patients with coronary syndromes, either as salvage therapy or as a bridge to surgery [71].

As a result, evidence consistently supports its safety and effectiveness in high-risk patients with acute post-myocardial infarction MR, moderate secondary MR, advanced heart failure, hypertrophic cardiomyopathy, and MAC, as well as in carefully selected candidates for combined structural interventions (M-TEER and transcatheter aortic valve implantation, M-TEER and tricuspid-TEER, or M-TEER and left atrial appendage closure) [72]. Additionally, TEER may represent a viable alternative to repeat surgery in appropriately selected congenital heart disease when performed by experienced multidisciplinary teams [73]. Across these settings, successful MR reduction is associated with meaningful symptomatic improvement and, in several scenarios, with improved survival compared with conservative therapy.

It is worth noting that M-TEER reshapes valve anatomy and complicates the development of additional treatment strategies. Unsuccessful procedural result or recurrent MR should be addressed with redo M-TEER as the preferred treatment approach, followed by mitral valve surgery (repair preferred over replacement) or alternative transcatheter therapies, such as the Amplatzer Vascular Plug [74]. Medical therapy should be considered only when further intervention is not appropriate.

#### 3.1.4. Contemporary Transcatheter MV Repair Techniques

Percutaneous left ventriculoplasty encompasses transcatheter procedures that accomplish direct, physical reverse remodeling of the LV by excluding nonviable scar/aneurysmal tissue [75]. The AccuCinch Ventricular Restoration System (Ancora Heart, Santa Clara, CA, USA) consists of anchors, radiopaque sliders, locks, and a cable that connects the anchors to the endocardial surface of the basal LV wall. It is delivered through the femoral artery and the aortic valve retrogradely into the LV under fluoroscopic and echocardiographic guidance. The anchors and sliders are placed in series below the mitral annulus in the basal-to-mid-LV wall. This not only reduces wall stress but also approximates the papillary muscles and reduces mitral leaflet tethering, leading to improved LV systolic performance and SMR reduction. Initial experience with the AccuCinch was described in a multicenter, single-arm, prospective study in 19 patients with HF and SMR (CorCinch-FMR). Preliminary results showed a 40% reduction in LV systolic and diastolic volumes, a reduction in MR, and improvements in LVEF, NYHA class, and quality of life at 6 months.

Transcatheter chordae repair devices have attracted much attention due to their lower trauma and better compliance with the physiological and anatomical structure [76]. Latib A. et al. [77] described the first in-human transcatheter mitral chordal repair in a 55-year-old patient with severe PMR due to P2 flail. The NeoChord NeXuS system was inserted transseptally into the left atrium via transfemoral venous access with a 28-F delivery catheter, and the artificial chords anchor at the head of the intended papillary muscle. This technology respects the MV geometry, can mimic the native chordal lengths, adds the advantage of beating heart implantation and chord tensioning, and has the potential to allow for additional surgical and transcatheter procedures if necessary [78].

### 3.2. Transcatheter MV Replacement

Early experience suggests that TMVR should be an alternative option based on MV anatomy. TMVR is divided into three types: (1) valve-in-valve (MViV) for severe MV disease, (2) valve-in-ring (MViR) for failed surgical repairs, and (3) valve-in-mitral annular calcifications (ViMAC) for mitral valvular disease with severe MAC and poor surgical criteria [79,80]. The wider range of approaches taken in TMVR system design reflects the many challenges faced for a successful device [6].

#### 3.2.1. Mitral Valve-in-Valve Implantation

**Figure 6:**
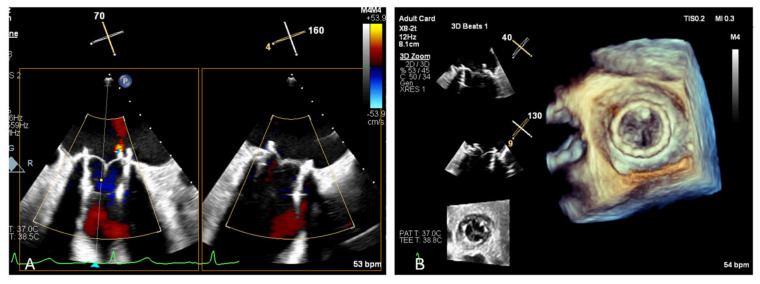
Transcatheter Mitral Valve-in-Valve implantation of a SAPIEN valve in a patient with a dysfunctional biological mitral valve. Interventional 3D-TEE (**A**). X-plane after SAPIEN valve deployment. (**B**) 3D zoom from left atrium perspective with multiplanar reconstruction.

MViV has emerged as an effective alternative to redo surgical mitral valve replacement in high-risk patients, demonstrating favorable procedural and clinical outcomes [81]. The greatest clinical experience has been with the balloon-expandable Edwards SAPIEN platform, including the SAPIEN, SAPIEN XT, and SAPIEN 3 valves (Edwards Lifesciences, Irvine, CA, USA) (Figure 6). Additional transcatheter heart valve (THV) systems used include INOVARE, MyVal, Lotus, and Direct Flow [82,83]. In MViV procedures, oversizing relative to the true internal diameter of the surgical bioprosthesis is generally recommended to optimize anchoring and reduce the risk of valve embolization in the setting of higher mitral closing pressures. However, excessive oversizing should be avoided, as it increases the risk of leaflet distortion, valve thrombosis, and reduced long-term durability [83].

In a retrospective cohort study, Won et al. [82] compared mViV implantation with redo surgical mitral valve replacement (rSMVR) for failed bioprosthetic mitral valves. Although both approaches achieved comparable 30-day procedural success, rSMVR was associated with superior long-term survival and greater hemodynamic durability among patients considered suitable surgical candidates by a multidisciplinary heart team.

#### 3.2.2. Mitral Valve-in-Ring

MViR procedures are more complex than MViV interventions due to substantial heterogeneity in annuloplasty ring design and composition. Identification of the implanted ring based not only on fluoroscopic data but mainly on operative reports and manufacturer specifications is critical for accurate procedural planning. Rings are broadly categorized as (Ι) complete or incomplete; and (ΙΙ) rigid, semirigid, or flexible. For procedural assessment, all available data should be carefully evaluated to successfully perform valve implantation, including the ring’s capacity to achieve circular deformation, its ability to provide stable valve anchoring, overall ring dimensions, and degree of radiopacity [84].

#### 3.2.3. Mitral Valve-in-MAC

TMVR is indicated for MAC-related mitral stenosis (MS), mixed MS/MR, and MR that is unsuitable for TEER. However, several challenging anatomic features complicate TMVR in MAC-associated MV disease [85].

A small or eccentric mitral annulus can increase the risk of device under-expansion during TMVR. Inadequate MAC severity may also result in valve migration, especially with balloon-expandable valves. A CT-based MAC scoring system evaluates calcium thickness, annular involvement, trigone calcification, and leaflet calcification. Score value ≤6 is associated with a higher risk of valve embolization or migration [86]. In addition, factors such as a narrow aortomitral angle, septal hypertrophy, a long anterior mitral leaflet, an eccentric annulus, and a small LV end-diastolic diameter increase the risk of left ventricular outflow tract obstruction (LVOTO) [87,88].

Several valve systems are currently used or under investigation for TMVR in MAC patients. Balloon-expandable valves such as the SAPIEN series and MyVal are commonly used off-label and rely on radial force and annular calcification for anchoring. The SAPIEN M3 system adds a docking mechanism that encircles the native mitral leaflets to create a secure landing zone for implantation. Dedicated self-expanding valves include the Tendyne valve, which consists of a trileaflet porcine pericardial valve attached to an outer frame and tethered to the ventricular apex for stability. Other investigational systems include the Intrepid valve, which uses an outer fixation frame and an inner bovine pericardial valve; the AltaValve system, with a supra-annular atrial anchoring design to reduce LVOTO; and the Cephea valve, which anchors via axial compression across the mitral annulus. These newer devices aim to improve sealing, reduce paravalvular leak, and minimize complications associated with MAC anatomy [85]. More than 30 dedicated TMVR devices are under development with novel deployment approaches and insertion, folding, fixation, and sealing mechanisms [89].

Patients with severe MAC, particularly those presenting with mixed MV disease, remain among the most challenging cases to treat. The rationale for applying lithotripsy in calcific MV disease is to create controlled fractures within the annular and leaflet calcium deposits, thereby promoting annular expansion and alleviating leaflet restriction [90]. The increased compliance of the valvular and annular structures reduces the risk of barotrauma and minimizes the potential for exacerbation of MR resulting from leaflet tears occurring at the junction between calcified and non-calcified tissue. The first published case in humans was reported by Eng et al. [91] in a patient with calcific MAC, using lithotripsy balloons in parallel, followed by mitral balloon valvuloplasty.

Furthermore, the hybrid transcatheter approach offers advantages, including resection of the anterior leaflet, septal myectomy, and reinforcement of the valve skirt. Novel interventional techniques such as BATMAN (Balloon-Assisted Translocation of the Mitral Anterior Leaflet) and transcatheter electrosurgical procedures should be incorporated into clinical practice to mitigate the risk of fatal LVOTO [85].

BATMAN was associated with high technical success and effectiveness in preventing LVOTO and appeared to be safe in MViR and MViV procedures, whereas adverse events were higher with ViMAC [85]. Future studies should focus on careful patient selection, as the advantages of technology have increased exponentially.

#### 3.2.4. Transcatheter Electrosurgery

There are limited treatment options for managing failed M-TEER, including redo M-TEER, high-risk surgery, or medical therapy. Residual MR and poor leaflet tissue quality may limit the feasibility of redo M-TEER. Transcatheter electrosurgery is a family of procedures that use radiofrequency energy to vaporize, traverse, or lacerate tissue [92]. These techniques are mainly used to facilitate TMVR. Electrosurgical laceration and stabilization of failed Mitraclip(s) (ELASTACLIP) is a novel technique for cutting the anterior MV leaflet to create a single orifice before TMVR [93]. Additionally, intentional laceration of the anterior mitral leaflet to prevent outflow obstruction (LAMPOON) is a transcatheter electrosurgical technique developed to mitigate neo-LVOTO risk prior to TMVR [94]. SESAME (SEptal Scoring Along Midline Endocardium) mimics surgical myotomy in order to reduce the hypertrophic bulge. It has been used in patients with symptomatic LVOTO or to create space to facilitate TMVR or transcatheter aortic valve replacement [95].

***Future Perspectives with Robotic-Assisted and Video-Assisted Minimally Invasive Surgery***

Continued refinements in robotic mitral surgery improve precision and reduce trauma, using smaller incisions and endoscopic techniques, leading to a favorable outcome for patients with MR. Robotic and totally endoscopic MV surgery represent effective minimally invasive treatment strategies for patients with MR. Both approaches have demonstrated excellent short- and mid-term clinical outcomes when performed in experienced centers. Compared with endoscopic surgery, robotic MV surgery has been associated with shorter hospital length of stay and a lower incidence of neurological complications, albeit at the expense of longer cardiopulmonary bypass times. Further studies are warranted to better define the optimal patient selection criteria and to establish the long-term durability and clinical outcomes of these evolving surgical techniques [96].

Furthermore, questions have arisen regarding the role of AI in predicting MVr success and durability. A recent study of Malik et al. [97] demonstrated that integrating AI/ML models into MV repair practice substantially improves the surgeon’s ability to predict patients’ outcomes and the durability of favorable results. Surgeons have the potential to surpass traditional methods by enabling personalized predictions and optimized surgical strategies. To enhance these achievements, Munafo et al. [98] recently presented, via deep learning, the MitraClip device automated localization in 3D TEE. This is the first attempt to automate device detection in an extremely complex case using intraoperative 3D TEE images acquired during the M-TEER procedure in a controlled in vitro simulation environment.

The field is moving toward more personalized, safer, and effective minimally invasive therapies by combining advances in imaging, device technology, and procedural techniques. The 2025 ESC guidelines introduce a revised classification of MR etiology that better reflects the underlying pathophysiologic mechanisms and provides more precise anatomical characterization to inform therapeutic decision-making. In this context, multimodality cardiovascular imaging plays a pivotal role by providing a comprehensive assessment of valvular anatomy, disease pathophysiology, and the global cardiac response to MR, thereby facilitating optimal patient selection and treatment planning. New innovations expand treatment options to a broader range of patients, including those previously considered high-risk for surgery. To this direction, the multidisciplinary Heart Team has a crucial role leading the important discussion on the evaluation and treatment decisions for patients with mitral valve disease. Continued research, technical refinement, and device innovation are mandatory.

## Data Availability

The original contributions presented in this study are included in the article. Further inquiries can be directed to the corresponding author.

## Abbreviations

The following abbreviations are used in this manuscript:

MR	mitral regurgitation
HF	heart failure
PMR	primary mitral regurgitation
SMR	secondary mitral regurgitation
M-TEER	mitral transcatheter edge-to-edge repair
MV	mitral valve
TMVR	transcatheter mitral valve replacement
LV	left ventricle
LA	left atrium
MAC	mitral annular calcification
TTE	transthoracic echocardiography
EROA	effective regurgitant orifice area
TEE	transesophageal echocardiography
3D	three-dimensional
CMR	cardiovascular magnetic resonance
LGE	late gadolinium enhancement
PET	positron emission tomography
GDMT	guideline-directed medical therapy
SGLT2i	sodium-glucose cotransporter 2 inhibitor
CRT	cardiac resynchronization therapy
LVEF	left ventricular ejection fraction
LVESD	left ventricular end-systolic diameter
MVr	mitral valve repair
ePTFE	expanded polytetrafluoroethylene
MVR	mitral valve replacement
MVA	mitral valve area
HTx	heart transplantation
MViV	mitral valve-in-valve
MViR	mitral valve-in-ring
ViMAC	valve-in-MAC
THV	transcatheter heart valve
rSMVR	redo surgical mitral valve replacement
MS	mitral stenosis
LVOTO	left ventricular outflow tract obstruction
BATMAN	balloon-assisted translocation of the mitral anterior leaflet
ELASTACLIP	electrosurgical laceration and stabilization of failed Mitraclip(s)
LAMPOON	laceration of the anterior mitral leaflet to prevent outflow obstruction
SESAME	septal scoring along midline endocardium
